# Microsatellite instability and intratumoural heterogeneity in 100 right-sided sporadic colon carcinomas

**DOI:** 10.1038/sj.bjc.6600474

**Published:** 2002-08-12

**Authors:** C Chapusot, L Martin, A M Bouvier, C Bonithon-Kopp, A Ecarnot-Laubriet, D Rageot, T Ponnelle, P Laurent Puig, J Faivre, F Piard

**Affiliations:** Service d'Anatomie Pathologique CHU Dijon, 21079 Dijon, France; INSERM EPI 01-06, IFR 100 Faculté de Médecine, 21079 Dijon, France; U 490, Laboratoire de Toxicologie Moléculaire, 75006 Paris, France

**Keywords:** colon carcinoma, right-sided colon, carcinogenesis, microsatellite instability, mismatch repair, intratumoural heterogeneity

## Abstract

Microsatellite instability has been proposed as an alternative pathway of colorectal carcinogenesis. The aim of this study was to evaluate the interest of immunohistochemistry as a new tool for highlighting mismatch repair deficiency and to compare the results with a PCR-based microsatellite assay. A total of 100 sporadic proximal colon adenocarcinomas were analysed. The expression of hMLH1, hMSH2 and hMSH6 proteins evaluated by immunohistochemistry was altered in 39% of the cancers, whereas microsatellite instability assessed by PCR was detected in 43%. There was discordance between the two methods in eight cases. After further analyses performed on other tumoural areas for these eight cases, total concordance between the two techniques was observed (Kappa=100%). Our results demonstrate that immunohistochemistry may be as efficient as microsatellite amplification in the detection of unstable phenotype provided that at least two samples of each carcinoma are screened, because of intratumoural heterogeneity.

*British Journal of Cancer* (2002) **87**, 400–404. doi:10.1038/sj.bjc.6600474 www.bjcancer.com

© 2002 Cancer Research UK

## 

Currently, there is accumulating evidence that some colorectal carcinomas have a different profile from others. Two apparently independent mechanisms of instability are recognised in colorectal cancer: chromosomal instability and microsatellite instability. The first group is characterised by allelic losses (LOH for loss of heterozygosity), chromosomal amplifications, and translocations in colorectal cancer cells. The second group is defined by an unstable phenotype (microsatellite instability (MSI) or replicative error (RER) positive) determined by genetic or epigenetic alterations of MisMatch Repair (MMR) genes. This phenotype is one of the major characteristics of tumours developing in genetically predisposed Hereditary Non-Polyposis Colorectal Cancer (HNPCC) patients (Aaltonen *et al*, 1993; Aaltonen, 1998). It is also involved in the occurrence of approximately 10 to 15% of sporadic colorectal carcinomas.

Colorectal carcinomas with MSI phenotype display particular clinical, pathological and prognostic features and their susceptibility to chemotherapy seems to be different (Branch *et al*, 1995; Boland, 1996; Claij and Riele, 1999; Elsaleh *et al*, 2000). Thus the determination of the molecular status of the tumour is a potentially important factor in the clinical and therapeutic management of these patients. A reliable PCR-based microsatellite instability test can be used to identify tumours with defective MMR, but this is time consuming and requires the services of a molecular genetics laboratory. Immunohistochemistry can now be performed (Thibodeau *et al*, 1996), and this technique could be a useful alternative strategy to molecular biology.

The aim of this study was to evaluate the interest of immunohistochemistry (IHC) as a new tool for detecting mismatch repair deficiency and to compare the results with a PCR-based microsatellite assay.

## PATIENTS AND METHODS

### Patients and samples

One hundred and eight proximal colon carcinomas, resected in Dijon University Hospital between September 1996 and November 2000, were included in this study. They were defined as proximal to the splenic flexure (Ponz de Leon *et al*, 1992). Eight cases were excluded (three men and five women) since DNA amplification was not possible. Thus a total of 100 proximal adenocarcinomas were analysed. There were 41 males and 59 females. No patient had HNPCC syndrome.

### Immunohistochemistry (IHC)

The MMR protein status was determined by an immunohistochemistry assay using a super sensitive immunodetection system (Biogenex, San Ramon, CA, USA) and Amino Ethyl Carbazol as chromogen. Five μm-thick sections of 10% formalin-fixed, paraffin embedded cancer slides were first placed in 10 mM citrate buffer (pH 6) and autoclaved for 5 min for antigen retrieval. The tissue sections were then incubated for 1 h with the primary monoclonal antibodies hMLH1 (1/100, Pharmingen, San Diego, CA, USA), hMSH2 (1/100, Pharmingen) and hMSH6 (1/800, Transduction Laboratories, San Diego, CA, USA). All the results were scored without knowledge of microsatellite instability status. Carcinomas were considered to demonstrate inactivation of the MMR system when there was complete absence of a detectable nuclear signal of neoplastic cells for at least one of the proteins (Fink *et al*, 1997). Intact nuclear staining of adjacent non neoplastic epithelium or of infiltrating lymphocytes was used as internal positive control and was required for adequate evaluation.

### Microsatellite amplification

For 56 cases, the DNA was extracted from freshly frozen tissue samples from a different area from the one employed for the IHC assay. For 44 cases, DNA was extracted from the paraffin embedded tissues used for the IHC. The DNA was extracted after macrodissection in order to reduce contamination by non-neoplastic inflammatory and stromal cells. The adjacent normal mucosa was sampled separately and used as a control. Two mononucleotide microsatellite markers (BAT26 and BAT25) were first used to evaluate the genomic instability of each tumour. When the two markers gave conflicting results three other dinucleotide microsatellites D2S123, D5S346, D17S250 were then amplified. According to the definition of Boland *et al* (1998), if at least two of these five loci were modified the cancer was considered as unstable. PCR was carried out in a 50 μl reaction volume containing 100 ng of genomic DNA, 8 pmole of each primer with one primer of each pair fluorescently labelled for allele detection (Life Technologies SARL, France). The PCR was performed on an Omn-E Hybaid thermocycler. Each PCR product was mixed with 0.5 μl Genscan 500 TAMRA size standard (Applied Biosystems, France) and 18.5 μl formamide and denatured for 5 min at 95°C. The PCR fragments were separated using an ABI Prism 310 instrument (Applied Biosystems) and further analysed by using GeneScan and Genotyper software (Applied Biosystems). Instability was evaluated by the presence of new PCR products after cancer DNA amplification that were absent in PCR products of the corresponding normal DNA amplification. Microsatellite status was assessed without knowledge of the IHC results.

### Additional analysis

When the results of the two techniques were divergent, immunohistochemistry and microsatellite amplification were performed again. The IHC analysis was carried out on two to five other blocks per carcinoma and microsatellites were amplified from the DNA extracted after macrodissection, taking into account the immunostaining pattern.

### Histopathological analysis

The haematoxylin-eosin stained slides were reviewed by two pathologists who were not aware of the MSI status. All the cancers were adenocarcinomas. Differentiation grading was based on the formation of glands: in well-differentiated cancers, more than 90% of the tumour cells formed glands; moderately-differentiated 30 to 90%; poorly-differentiated less than 30%. Mucin secretion was categorised as absent (no mucin production), focal intracytoplasmic (<50%), focal extracellular (<50%), while mucinous cancers corresponded to carcinomas with areas of extracellular mucin greater than 50%. Lymphocytic stromal reaction was classified in sheets, aggregates or Crohn's like lymphoid reaction which was defined as pronounced lymphoid reaction to the tumour composed of lymphoid follicles with germinal centres at the tumour edge (Kim *et al*, 1994; Jass *et al*, 1998). The TNM system of UICC was used for tumour staging (Hermanek *et al*, 1997).

### Statistical analysis

The associations between the clinicopathological features of the cancers and the MSI status were evaluated with the Pearson Chi-square test of heterogeneity. The concordance between the two IHC and PCR-based assay outcomes was analysed with the Kappa-statistic measure. Analysis was carried out using the STATA software package (Stata software 1984–99. Stata Corporation, 702 University Drive, East College Station, Texas, TX 77840, USA).

## RESULTS

### Clinical and pathological characteristics

The mean size of tumours was 33.1±2.6 cm^2^. Of the 100 specimens of right-sided colon cancers, 10 were poorly differentiated. Mucin secretion was observed in 42% of the cases. It was focal intracytoplasmic in 27 cases and focal extracellular in 11 cases. Four mucinous carcinomas were diagnosed. Peritumoral lymphocytic infiltration was seen as nodules or follicles in 70% of the cases. According to the TNM system of staging, eight carcinomas were stage I, 60 stage II, 24 stage III and eight stage IV.

### Immunohistochemistry (IHC) results (Table 1)

**Table 1:**
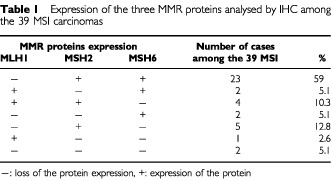
Expression of the three MMR proteins analysed by IHC among the 39 MSI carcinomas

IHC analysis showed no nuclear staining of carcinoma cells for at least one of the three MMR proteins in 39 of the 100 cases. Of these, 74.4% (29 cases) presented a loss of the signal of only one protein, 20.5% (eight cases) presented a loss of the expression of two proteins and 5.1% (two cases) a loss of the expression of all three proteins.

The link between the clinicopathological features of the 100 right-sided colon adenocarcinomas and the MSI status evaluated by IHC was assessed (Table 2

**Table 2:**
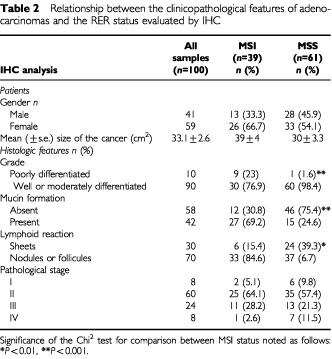
Relationship between the clinicopathological features of adenocarcinomas and the RER status evaluated by IHC

). The mean size of the cancers tended to be greater in the MSI cancers: 39 cm^2^±4 against 30 cm^2^±3.3 among the 61 stable cancers (NS). The MSI carcinomas were more likely to be poorly differentiated than the stable (MSS) cancers: 23.1% of the cancers showing loss of MMR-protein expression were poorly differentiated whereas only 1.6% of the cancers with normal nuclear expression of MMR proteins were poorly differentiated (*P*=0.001). A mucinous component was more frequently observed in the MSI cancers: 69.2% of the MSI cancers were characterised by mucin production whereas 24.6% of the stable cancers presented mucin secretion (*P*<0.001). Moreover, 84.6% of the MSI cancers had a lymphoid stromal reaction (follicles or nodules) while this same stromal reaction was observed in 60.7% of the MSS cancers (*P*=0.01).

### Microsatellite instability analysis

The PCR-based assay performed independently from the IHC analysis on the 100 carcinomas revealed microsatellite instability phenotype (MSI) in 43 cancers. Both BAT26 and BAT25 loci were involved in genomic instability in 38 cases. In the last five samples, BAT25 and BAT26 loci amplifications gave contradictory results. So three other microsatellites were investigated. In one case BAT26 was unstable and BAT25 stable but D5S346 and D17S250 had also shifted, which led us to consider this case as unstable (MSI). For the other four cases, BAT26 was stable and BAT25 unstable, but at least one of the other loci was modified; these cancers were also considered as unstable (MSI). As regards the other 57 cancers, no instability was detected at any locus analysed. Only a shift for the BAT26 locus in one tumour was noticed; the other four loci were unchanged. Thus, the tumour phenotype was regarded as stable (MSS).

### Concordance between IHC and PCR to assess the MSI status

When BAT25 and BAT26 amplifications were performed on DNA extracted after macrodissection from an area of the cancer on which IHC had been done, the results of the MSI status were the same in 43 of the 44 cases: 19 were unstable after PCR (MSI) and showed a loss of at least one MMR protein (MSI), 24 were stable (MSS) and presented normal immunostaining of the three proteins (MSS). The divergent case presented normal expression of the three MMR proteins but microsatellite instability after PCR. Thus the IHC method was nearly as efficient as the molecular method when the investigations were performed on the same tissue sample (Kappa=95%).

When DNA was extracted from a frozen sample taken from a different area from the one used for the IHC study, there was agreement between the two methods for MSI status in 49 out of 56 cases: 18 were unstable after PCR (MSI) and showed a loss of at least one MMR protein (MSI), 31 were stable (MSS) and presented normal immunostaining for the three proteins (MSS). There was disagreement between the two tests in seven cases: five cases were characterised by normal expression of the three MMR proteins but microsatellite instability after PCR. The last two cases had lost the expression of hMLH1 or of both hMLH1 and hMSH2 but no microsatellite instability was detected by PCR. Thus, the concordance between the two techniques can be considered as moderate when the analysis is performed on two different tissue samples (Kappa=73.7%).

Thus, at the end of the first round of analyses, the results from the two techniques in a total of eight cases were discordant.

### Secondary analyses of the 8 ‘discordant cases’

For one case, for which DNA was extracted from the paraffin embedded sample, a focal lack of hMLH1 staining was discovered when IHC was performed on the same tissue sample and on other additional samples. Nevertheless, this event was more frequent when DNA was extracted from a frozen sample that was different from the paraffin embedded sample used for IHC. In fact, for the seven other discordant cases, in which the two techniques were performed on two different areas of the tumour, we noticed heterogeneous staining on the supplementary blocks. However, the same patterns (five MMR efficient and two MMR deficient) were found again when IHC was performed on the original tissue sample. The different areas defined by immunohistochemistry (regions of expression or of extinction) were separated and DNA was extracted. We again performed amplifications of BAT25 and BAT26 loci on the DNA samples. The results of the PCR analysis were given without knowledge of the immunohistochemistry data. In all cases, microsatellite instability was detected with both markers in the areas of loss of IHC staining. In the same way, no microsatellite instability was noticed on the DNA samples extracted from areas with normal expression of the three proteins. Thus, the two methods of evaluation of the unstable status were equally informative when they were performed on the same area.

### Pathological features of the eight heterogeneous cancers

In the eight heterogeneous cases, the foci showing mismatch repair deficiency were not usually limited to minor subclones. Heterogeneity concerned either a large area in a block or larger regions in different blocks of the same cancer. The heterogeneity involved one to three MMR proteins: in two cases the three proteins, in four cases two proteins (hMLH1 and hMSH6) and in two cases one protein (either hMLH1 or hMSH6). Sometimes the foci of MMR deficiency were superimposed, elsewhere mutually exclusively expressed.

The eight adenocarcinomas were located in the caecum (four), in the ascending colon (three) and in the transverse colon (one). One cancer was poorly differentiated whereas the seven remaining cases were moderately or well-differentiated. Mucin was observed in four cancers and a lymphoid reaction as nodules or follicles was seen in seven cases. Seven patients were stage II and one stage IV.

## DISCUSSION

In recent years, much progress has been made in the understanding of the molecular genetics of colorectal carcinoma (Fearon and Vogelstein, 1990). It is now widely admitted that microsatellite instability is an alternative genetic pathway in colorectal carcinogenesis. Testing tumours for the presence of underlying MMR deficiency requires the services of a molecular genetics laboratory and cannot be performed routinely. The evaluation of MMR deficiency by the IHC technique is based on the loss of nuclear detection of at least one of the MMR proteins in the cancer cells. It has been suggested that immunohistochemistry could represent an alternative strategy to molecular biology (Thibodeau *et al*, 1996). This study indicates that IHC detection of the MMR proteins may be as efficient as a PCR based assay for screening MMR deficiency in certain conditions.

Analysing a total of 100 carcinomas, we have confirmed the association between MSI positivity and the loss of protein expression reported in other studies (Thibodeau *et al*, 1996; Dietmaier *et al*, 1997; Cawkwell *et al*, 1999). After the first analysis, immunochemistry of hMLH1, hMSH2 and hMSH6 proteins identified 39 MSI carcinomas. This percentage (39%) is slightly higher than those previously reported in proximal sporadic colon carcinomas which varied between 23.1 and 33% (Kim *et al*, 1994; Cawkwell *et al*, 1999; Chao *et al*, 2000; Hemminki *et al*, 2000). These studies were only performed with hMLH1 and hMSH2 whereas in the present series, the use of the hMSH6 antibody enabled us to identify 10.3% of the MSI cases (i.e. 4% of the 100 cases). As recently reported (Thibodeau *et al*, 1998), we also noted a preponderance of hMLH1 abnormalities over hMSH2 defects (overall 5 : 1) and over hMSH6 deficiency (overall 3 : 1). As the loss of hMLH1 expression correlates with hypermethylation of its promoter (Kane *et al*, 1997; Herman *et al*, 1998), we hypothesised that an excess of hMLH1 abnormalities could be produced since the hMSH2 promoter is less likely to be inactivated via this mechanism (Cawkwell *et al*, 1999). One patient had a lack of expression of hMLH1 and hMSH2, suggesting somatic mutations of both of these genes during tumour progression, or an inherited defect in one gene and a somatic alteration of the other.

An interesting point of the study was the discovery of a discrepancy between the two methods according to the area of analyses. When the DNA was extracted from the carcinomatous area on which IHC had been performed, one (2.3%) IHC negative test (i.e. MMR deficiency missed) was found out of the 44 cases of the first group. When IHC was performed again on this sample, we noticed that nuclear immunostaining was not diffuse but focal with a regional loss of hMLH1. In this case, the high sensitivity of PCR assay was able to detect and to amplify instability even though it concerned only a small number of cancerous cells. Furthermore, the paraffin block had been exhausted for DNA extraction. Then, we can hypothesise that this enabled us to discover intratumoural heterogeneity of hMLH1 expression between the first section and the following ones. In the same way, five IHC negative tests (8.9%) were observed when the DNA was extracted from carcinomatous areas different from those on which IHC had been carried out (56 cases) (second group). Two PCR negative tests (3.6%) also showed a loss of the IHC signal. We first interpreted this discrepancy as the result of the use of different techniques. In the second round of analyses, this fact was explained by the presence of intratumoural heterogeneity.

Regional variability of hMSH6 expression was noticed in five of the eight heterogeneous cases. This could be interpreted as a secondary event acquired after hMLH1 deficiency. This fact had previously been noticed by Samowitz and Slattery (1999) concerning the focal presence of a frameshift mutation in four coding mononucleotide repeats (MSH6, IGFIIR, BAX and MSH3). According to the authors, this was consistent with a somewhat later time of acquisition of these mutations. Nevertheless, this explanation is not valid for the cases where hMSH6 was the only focal deficient protein. This alteration could not be interpreted as a germ line mutation (since the lack of expression was focal), but as an independent event of the hMLH1 status, or as the consequence of the alteration of another MMR protein not studied in this work.

The contemporary presence of areas with normal staining for the three MMR proteins (and no instability of BAT25 and BAT26) and regions with loss of MMR protein expression (and microsatellite instability at the molecular level) is more difficult to explain in the light of our present knowledge on the biological and genetic characteristics of these tumours. These results raise the question of the existence of genuine mixed tumours. In order to determine the existence of loss of heterozygosity, we genotyped different areas of the tumours after macrodissection. For this purpose, five microsatellite loci mapped to 18q were typed on normal and tumour DNA. Unfortunately, we were unable to determine reliable genotyping in tumour DNA extracted from paraffin embedded tissue sections. This negative result is probably due to the low quality of the DNA extracted from formalin fixed, paraffin embedded samples (Coulet *et al*, 2000). So we were not able to determine whether these tumours showed partly chromosomic instability. MSI positive tumours have particular clinical and histopathological features (Lothe *et al*, 1993; Thibodeau *et al*, 1993, 1998; Kim *et al*, 1994; Risio *et al*, 1996; Rüschoff *et al*, 1997; Jass *et al*, 1998; Gafa *et al*, 2000). In our series, the different features associated with MSI status conformed with the characteristics described in the literature: the presence of a mucinous component, rather poor differentiation, a Crohn's like lymphoid stromal reaction and a trend towards a tumour of a larger size. These traits were compared with those of the eight cancers exhibiting heterogeneous MMR deficiency, and no significant characteristic allowed us to distinguish these ‘heterogeneous’ cancers from other MMR deficient cases.

Additionally, the IHC MMR-protein analysis would be helpful for the identification of a high-risk group of individuals having HNPCC syndrome. In contrast to microsatellite test, IHC identifies specifically either hMLH1, hMSH2, or hMSH6 as the underlying inactivated gene. Therefore it directs germline mutation analysis to one gene, and saves unnecessary analyses of other MMR genes in HNPCC patients.

In conclusion, the evaluation of MSI status can be accurately appreciated by both methods if at least two samples from different areas of each cancer are analysed. This raises the problem of sampling for giving an accurate evaluation of the unstable status of the cancer. It would be interesting to screen several samples of the 100 cancers analysed for MMR lack with the aim to evaluate the level of intratumoural variability. It would enable us to establish the minimum number of samples required for the most efficient evaluation of MMR deficiency.

Nevertheless, immunohistochemical analyses of two tissue samples with three antibodies would require at least six tissue sections for each tumour. Accordingly, the workload and costs for histopathology laboratories would be much greater. This is an important issue taking into account the possibility to introduce immunohistochemical analysis of MMR protein expression as a routine diagnostic test and would provide valuable management information in addition to the histopathological assessment of tumour stage and grade. This is all the more important since the predictive value of the MSI status in the good response to chemotherapy has recently been stressed (Elsaleh *et al*, 2000; Hemminki *et al*, 2000; Watanabe *et al*, 2001).
